# Brain Activity and Functional Coupling Changes Associated with Self-Reference Effect during Both Encoding and Retrieval

**DOI:** 10.1371/journal.pone.0090488

**Published:** 2014-03-07

**Authors:** Nastassja Morel, Nicolas Villain, Géraldine Rauchs, Malo Gaubert, Pascale Piolino, Brigitte Landeau, Florence Mézenge, Béatrice Desgranges, Francis Eustache, Gaël Chételat

**Affiliations:** 1 Inserm, U1077, Caen, France; 2 Université de Caen Basse-Normandie, UMR-S1077, Caen, France; 3 Ecole Pratique des Hautes Etudes, UMR-S1077, Caen, France; 4 CHU de Caen, U1077, Caen, France; 5 Université de Paris Descartes, Laboratoire Mémoire et Cognition, Institut de Psychologie, Paris, France; 6 Inserm, U 894, Centre de Psychiatrie et Neurosciences, Paris, France; Yale University, United States of America

## Abstract

Information that is processed with reference to oneself, i.e. Self-Referential Processing (SRP), is generally associated with better remembering compared to information processed in a condition not related to oneself. This positive effect of the self on subsequent memory performance is called as Self-Reference Effect (SRE). The neural basis of SRE is still poorly understood. The main goal of the present work was thus to highlight brain changes associated with SRE in terms of activity and functional coupling and during both encoding and retrieval so as to assess the relative contribution of both processes to SRE. For this purpose, we used an fMRI event-related self-referential paradigm in 30 healthy young subjects and measured brain activity during both encoding and retrieval of self-relevant information compared to a semantic control condition. We found that SRE was associated with brain changes during the encoding phase only, including both greater activity in the medial prefrontal cortex and hippocampus, and greater functional coupling between these brain regions and the posterior cingulate cortex. These findings highlight the contribution of brain regions involved in both SRP and episodic memory and the relevance of the communication between these regions during the encoding process as the neural substrates of SRE. This is consistent with the idea that SRE reflects a positive effect of the reactivation of self-related memories on the encoding of new information in episodic memory.

## Introduction

One's sense of self fundamentally depends on self-knowledge and memories of one's past experiences [1]–[3]. The processing of information in a self-referential manner, i.e. with reference to oneself, is known as Self-Referential Processing (SRP). SRP generally consists in linking implicitly or explicitly the information to-be-processed with pre-existent semantic self-knowledge and autobiographical memories (for review, [4]).

Numerous neuroimaging studies have been conducted to assess the underlying mechanisms of SRP, for example by asking participants to judge whether a trait adjective describes their personality. These studies consistently reported the involvement of cortical midline structures such as the medial prefrontal cortex (MPFC) extending to the anterior cingulate cortex (ACC) and the posterior cingulate cortex (PCC) [5]–[9]. While other regions are also implicated in specific aspects of the self, such as temporo-parietal areas in agency (i.e. the feeling of being causally involved in an action), cortical midline structures are thought to be involved in the “core self” in that they are crucial to the processing of self-referential stimuli even if they are differentially involved in the different self-related processes. In addition, the MPFC is considered as a key-component of the Self-Memory System [10] as it is activated regardless of the level of abstraction of information related to the Self (from general self-knowledge to specific autobiographical memories) while other structures would tend to be involved in specific conditions e.g. in relation to the recollection of autobiographical memories for the hippocampus and the posterior areas [11]–[14].

An extensive literature has demonstrated that SRP enhances subsequent memory performance. Rogers et al. [15] first reported that personality trait adjectives rated under a self-reference task were better recalled than words processed in non self-related conditions such as semantic processing. The positive effect of SRP on memory performance is called as Self-Reference Effect (SRE) and has been repeatedly observed since then [16]–[19]. However, little is known about the brain correlates of the positive effect of SRE. The few previous neuroimaging studies on SRE have consistently reported the involvement of the MPFC, especially the ventral MPFC, during either encoding or retrieval [20]–[23], sometimes extending to the anterior cingulate cortex [22] or together with other brain areas such as the hippocampus [23]. These results suggest that SRE may be related to changes mainly in cortical midline structures known to be involved in SRP, but also in the hippocampus known to be involved in episodic memory processes, during either encoding or retrieval.

However, no study to date has considered brain activity during both encoding and retrieval. Yet, this is crucial to understand the relative contribution of each process to SRE, i.e to know if improved memory performances of self-related information are more related to brain activity changes during the encoding vs the retrieval of this information. More specifically, as previous studies suggest the involvement of brain structures preferentially involved in self (MPFC), episodic memory (hippocampus), or both processes (PCC), it seems of particular interest to assess their relative involvement during encoding versus retrieval of self-related information and the role of their interaction in SRE. As SRE is thought to reflect a positive effect of the reactivation of self-related memories on the encoding of new information in episodic memory [4], we hypothesized that SRE at least partly results from a reinforced interaction between self-related and episodic memory networks, i.e. increased connectivity between these two overlapping brain systems, during encoding. As the most obvious overlapping area, the PCC lays in a strategic position and is thus thought to play a central role in reinforcing this interaction. The main goal of the present study was thus to highlight brain changes associated with SRE in terms of activity and functional coupling and during both encoding and retrieval. For this purpose, we used an fMRI event-related self-referential paradigm in healthy young subjects. We first identified the brain networks underlying SRP during both encoding and retrieval. Second, we investigated brain activity and functional coupling changes specifically associated with the encoding of self-reference information (successfully recalled in the subsequent retrieval task), and with the successful retrieval of self-reference information.

## Materials and Methods

### Participants

Thirty right-handed native French-speaking participants were included in our study (13 men; 17 women; age: 29.3±6.9 [19]–[40] years old; years of education: 13.5±2.9 [9]–[20]). Healthy subjects were enrolled in this study after detailed clinical and neuropsychological examinations. They were screened for the lack of abnormalities according to stringent inclusion/exclusion criteria including (1) normal somatic examination; (2) body mass index in the normal range; (3) no known vascular risk factor and smoking less than 10 cigarettes per day; (4) no alcohol or drug abuse; (5) blood pressure within normal limits; (6) no history or clinical evidence of neurological disease, dementia, or psychiatric disorder; (7) no current use of medication (except birth control pills, estrogen replacement therapy, and antihypertensive drugs); and (8) normal standard T1- and T2-weighted magnetic resonance imaging (MRI) scans as assessed by a medical doctor. All subjects had performance in the normal range (i.e., within 1.65 standard deviation of the normal mean for age) in all screening neuropsychological tests, i.e. in general intellectual function (Mini Mental State Examination [24] and Mattis Dementia rating scale [25]), verbal (RL-RI 16 [26]) and visual (BEM-144 Figure recall [27]) episodic memory, executive function (Stroop test [28]), visuospatial function (Rey Osterrieth Complex Figure Copy [29]), gestual praxis (imitation of four meaningless gestures, production of four symbolic gestures and four object utilization gestures), language (writing of 12 irregular words under dictation) and image naming (DO80 [30]). The tests were administered and scored by a neuropsychologist. No subject complained about his or her memory. Signed informed consent was obtained prior to participation. The study was approved by the local ethical committee (CPP Nord-Ouest III) and was carried out in line with the declaration of Helsinki.

### Data acquisition

#### Design and task

The fMRI event-related self-referential paradigm used in the present study was adapted from the paradigm used in previous publications on SRP and SRE [6], [7], [31]–[33]. The selected materials consisted in a list of 204 personality trait adjectives selected from 463 adjectives issued from a French language dictionary (http://atilf.atilf.fr/). The selection of adjectives was based on their familiarity and valence ratings obtained from a pre-experiment in samples of young and elderly individuals with low and high education. For the sake of the fMRI experiment, 6 lists of 24 adjectives were constituted from the 204 selected adjectives for the two runs per condition (n = 3) of the encoding session and 2 lists of 30 adjectives were constituted to be used as distractors in the retrieval session. The adjectives in the 8 lists (to be used in the different experimental conditions), were counterbalanced for familiarity, valence, and number of letters so that these parameters didn't differ between conditions. For the experiment, the selected adjectives were successively displayed to the subjects who had to indicate whether or not the adjective described either themselves (Self condition) or a celebrity (Other condition), or whether the adjective was positive or not (Semantic control condition). As in most previous studies assessing SRP, the semantic condition was used here as the control condition. Indeed, the “Other condition” appears to be a less appropriate control condition to assess self-related processes because it is thought to involve self-relevant processes [8], [20], [34], [35]. Indeed, theoretically, other's representation is closely related to our self-representation. Thus, according to the “simulation theory” for instance, individuals use their own experience to infer the mental states of others [36]–[38]. Moreover, neuroimaging findings assessing self- and other-reference processing revealed highly overlapping activation networks notably including the ventral and dorsal MPFC and the PCC ([33] for review [39]), as illustrated from the data of the present study (Figure S1). While all analyses were thus conducted comparing the self to the semantic conditions, the mean BOLD value was indicated in each cluster of interest for the Other condition as well for the sake of completeness.

**Figure 1:**
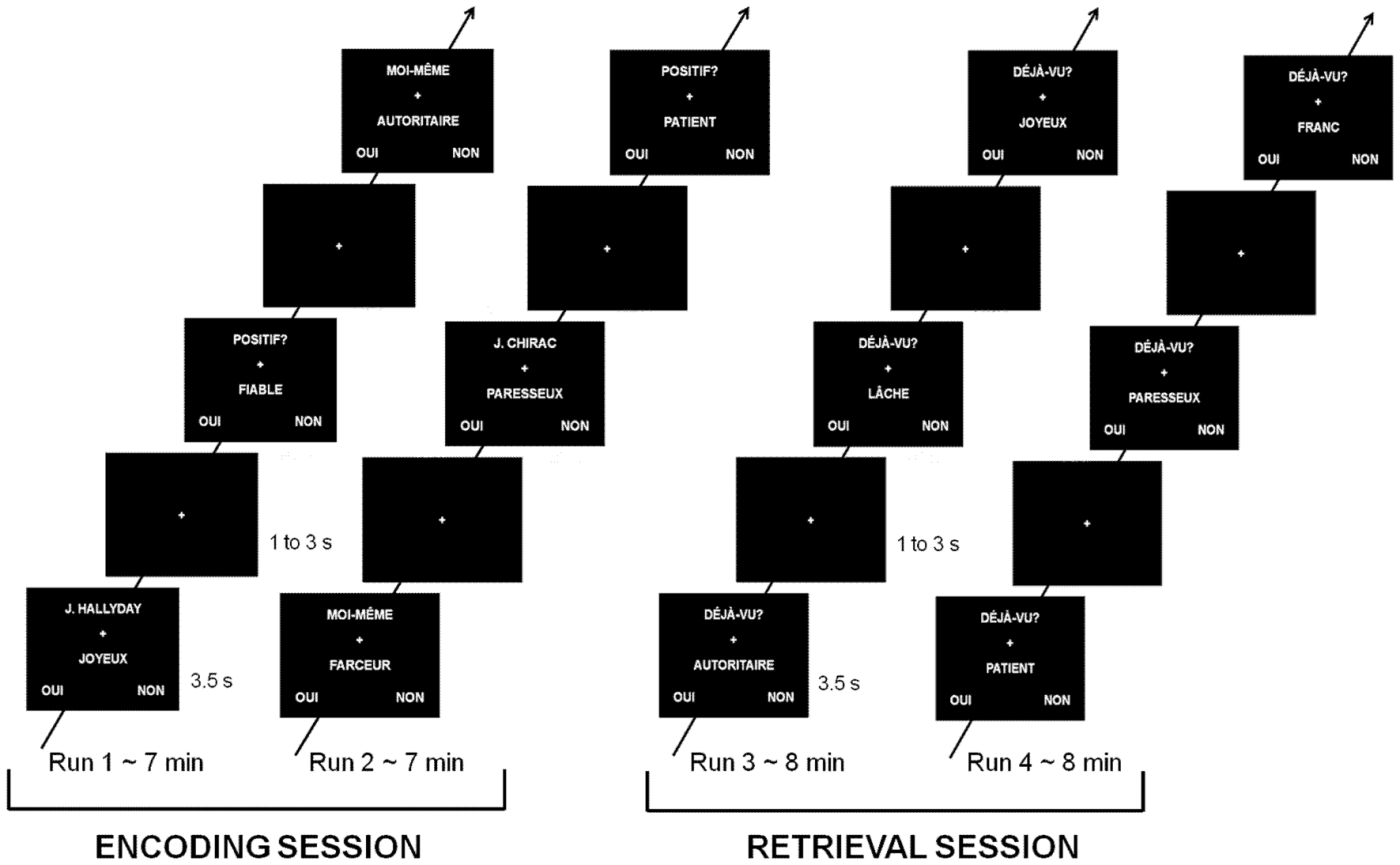
Design of the fMRI task with the encoding (left) and retrieval (right) sessions. Translations: MOI-MÊME = myself; POSITIF =  positive; DÉJÀ-VU? =  Old?; AUTORITAIRE  =  authoritarian; FIABLE  =  trustworthy; JOYEUX  =  happy; PATIENT  =  patient; PARESSEUX  =  lazy; FARCEUR  =  joker; LÂCHE  =  cowardly; FRANC  =  honest; OUI =  yes; NON =  no.

Subjects were not aware of the subsequent retrieval task. After a pre-experimental training session performed outside the scanner to familiarize the subjects with the task, subjects underwent two functional runs, each lasting about 7 minutes and including 72 stimuli with the same proportion of positive and negative adjectives (12 positive Self, 12 negative Self, 12 positive Other, 12 negative Other, 12 positive Semantic, 12 negative Semantic). Note that the adjectives presented during the training pre-experimental session were different from those presented during the scan, so that the adjectives used in the experiment were all presented for the first time during the encoding session. An overall view of the experiment is provided in Figure 1. Each adjective was presented on a screen for a duration of 3500 m, along with brief instructions on the nature of the process to perform (i.e. “Myself”, “J. Chirac” or “J. Hallyday”, and “Positive?”, corresponding to the Self, Other, and Semantic conditions respectively), followed by a fixation cross for 1000 to 3000 ms (mean: 2000 ms). In each trial, subjects had to answer Yes or No with their right or left index fingers on a two-button keyboard. Just after the encoding session, a surprise recognition task was proposed where they should indicate whether the adjectives have been already presented or not (during the previous encoding session). Each retrieval run lasted about 8 minutes and included 84 adjectives (30 new adjectives, 18 old Self, 18 old Other, 18 old Semantic; with the same number of positive and negative items in each category). As for the previous encoding session, adjectives were presented on a screen for 3500 ms, along with brief instructions on the nature of the task to perform (“Old?”), followed by a fixation cross of 1000 to 3000 ms (mean: 2000 ms), and subjects had to answer Yes or No with their right or left index fingers on a two-button keyboard. Adjectives from the first encoding run were presented in the first retrieval run, while adjectives from the second encoding run were presented during the second retrieval run. The order of conditions within each run was optimized using a Genetic Algorithm in order to enhance the detection of fMRI differences between the experimental conditions in the subsequent SPM statistical analyses [40]. Moreover, because adjectives for different conditions were issued from different lists, familiarity, valence and number of letters were rigorously counterbalanced across conditions. Note that the valence was counterbalanced and controlled for in the following analyses (see below). Lists of adjectives used for each condition, as well as the side of the Yes versus No answer on the keyboard were also counterbalanced across subjects. Items were displayed using the E-Prime software (Psychology Software Tools, Pittsburgh, PA) implemented within IFIS System Manager (Invivo, Orlando, FL).

#### Neuroimaging data acquisition

A Philips (Eindhoven, The Netherlands) Achieva 3.0 T scanner from the GIP Cyceron (Caen, France) was used for data acquisition. For each participant, a high resolution T1-weighted anatomic volume was first acquired using a 3-dimensional fast field echo (FFE) sequence (3D-T1-FFE sagittal), followed by a high-resolution T2-weighted spin echo anatomical acquisition (2D-T2-SE sagittal) and a non-Echo-Planar Imaging (EPI) T2 *volume (2D-T2 *-FFE axial). For the functional acquisition, eleven subjects (6 women and 5 men; mean age: 30±7.6) had an interleaved 2D T2 Star echo planar imaging (EPI) sequence (2D-T2 Star-FFE-EPI axial, TR = 2200 ms; TE = 35 ms; flip angle = 80°; 35 slices; slice thickness = 3.5 mm; no gap; matrix = 64×64; FoV = 224×224 mm; in-plane resolution = 3.5×3.5 mm; 185 volumes per run for the encoding session and 215 volumes per run for the retrieval session) and nineteen subjects (11 women and 8 men; mean age: 28.9±6.7) underwent an interleaved 2D T2 Star SENSE (SENSitivity Encoding) EPI sequence (2D-T2 Star-FFE-EPI axial, SENSE factor = 2; TR = 2382 ms; TE = 30 ms; flip angle = 80°; 42 slices; slice thickness = 2.8 mm; no gap; matrix = 80×80; FoV = 224×224 mm^2^; in-plane resolution = 2.8×2.8 mm^2^; 172 volumes per run for the encoding session and 199 volumes per run for the retrieval session). Note that in a previous study we showed that there was no significant differences between the two EPI sequences in SRP-related brain activity during encoding [41]. Moreover, all analyses presented here were performed both pooling data from the two EPI sequences together, and only using the SENSE EPI sequence obtained in 19 subjects (data not shown). The results were almost identical so that only the results from the entire sample (n = 30) will be presented here.

### Data analysis

#### Behavioral analysis

The percentage of recognized items (Hits/(Hits+Misses) x 100) was calculated for each encoding condition (Self and Semantic) according to the subjects' answers during the retrieval session. These two percentages were then each compared across subjects to the random level (i.e. 50%) using one-sample t-tests. In order to assess the benefit of self-reference encoding on memory performances (i.e. the SRE), the percentage for the Self condition was compared to those for the Semantic condition using a paired t-test. Statistical analyses were performed using the Statistica software (Statsoft®, Tulsa).

#### Neuroimaging analysis: pre-processing, brain activity and functional coupling analyses

fMRI data were pre-processed using the procedure detailed in Villain et al. [41]. In short, the EPI volumes were corrected for slice timing and realigned to the first volume. Data were then spatially normalized using a technique designed to reduce geometric distortion effects [41]. This procedure includes for each individual (1) a coregistration of the mean EPI volume, non-EPI T2*, T2, and T1 volumes; (2) a warping of the mean EPI volume to match the non-EPI T2* volume; (3) a segmentation of the T1 volume using the VBM 5.1 ‘Segment’ procedure with the International Consortium for Brain Mapping/Montreal Neurological Institute priors; (4) a normalization of the coregistered T1, EPI, and non-EPI T2* volumes using the parameters obtained from the segmentation of the T1 volume; and (5) a 8 mm FWHM smoothing of the EPI volumes.

Statistical analyses were conducted on functional images using SPM5 (Statistical Parametric Mapping software; http://www.fil.ion.ucl.ac.uk/spm/) and the general linear model approach on a voxelwise basis with a random effects model implemented with a two-level procedure. Based on the subjects' answers during the retrieval session, subsequently remembered versus forgotten adjectives were identified and the four experimental conditions of interest (Self Remembered, Self Forgotten, Semantic Remembered and Semantic Forgotten) were modeled as δ functions at each stimulus onset of the encoding session. In addition, four conditions of non interest in the encoding session (Other Remembered, Other Forgotten, Not Seen during Retrieval, No Response) and five conditions of non interest in the retrieval session (Other Remembered, Other Forgotten, Correct Reject, False retrieval, No Response), as well as the subjects' response time and the valence for each stimulus, were also modeled in order to get an accurate and reliable measure of first level noise estimates. The ensuing hemodynamic response was modeled by convolving these δ functions with a canonical hemodynamic response function. An “individual structural mask” was created for each individual to be used as an explicit mask in all individual (1^st^ level) analyses of the corresponding subject. This mask corresponded to the conjunction between the gray matter segment from the T1 volume (including only values >0.15) and the non EPI-T2* volume (including only values >0.05) of the subject. For the group (2^nd^ level analyses), a “group structural gray matter mask” (obtained from the mean gray matter segment and the mean non EPI-T2* volume of the group) or a “group functional mask” (see below and Figure S2) was used instead.

First, to highlight brain activity associated with SRP, the main effects of self and semantic judgments (independently from the retrieval of the items) were assessed individually for both the encoding and the retrieval sessions (1^st^ level analyses). For each session, the resulting individual contrast images were then entered into a second level analysis corresponding to a paired t-test between the two conditions (Self and Semantic). Second, to highlight brain activity associated with SRE, analyses were conducted in two steps as we were interested in highlighting differences across conditions within particular regions only so that a so-called “group functional mask” corresponding to these particular regions was first created (see Figure S2 for further details). More specifically, to highlight SRE-related brain activity, i.e. brain activity related to the successful encoding (or retrieval) of self compared to semantic items, we assessed the differences between the self and the semantic conditions only within brain regions associated with the successful encoding (or retrieval) of self-related information. We thus created, as a first step, “group functional masks” corresponding to brain activity associated with the successful encoding (or retrieval) of self-related information.

**Figure 2:**
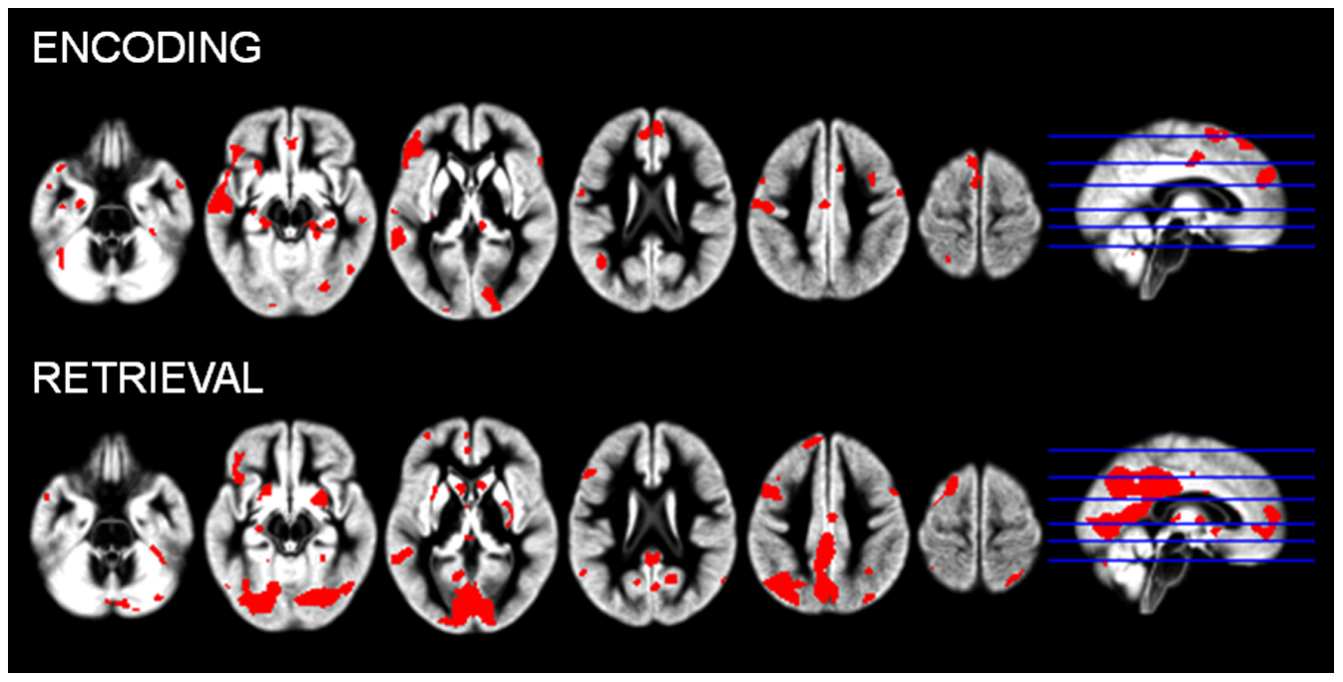
Brain activity changes associated with successful encoding and retrieval of self-referential information. Results during encoding (top line) and retrieval (bottom line) are displayed at p<0.05 uncorrected to be used as masks for analyses of SRE.

To create these group functional masks, individual images of the differences between ‘Self Remembered’ and ‘Self Forgotten’ items were obtained for both the encoding and the retrieval sessions in first level analyses, and these individual images were entered in one-sample t-tests for second level analyses. The results of these analyses were saved as binary images at uncorrected p<0.05. Two different functional masks were thus created (one for encoding and one for retrieval) (see Figure 2), and each was entered (as an inclusive mask) in the corresponding following analysis.

In a second step, corresponding to the main analyses of interest assessing the effects of SRE per se, individual images of the differences between ‘Self Remembered’ and ‘Semantic Remembered’ items were computed for both the encoding and the retrieval sessions in first level analyses, and resulting individual images were entered in paired t-tests for second-level analyses.

This two-step functional masking procedure allowed assessing, within the regions associated with the successful encoding (or retrieval) of self-related information, which ones are significantly different from those associated with the successful encoding (or retrieval) of items from the semantic condition.

**Figure 3:**
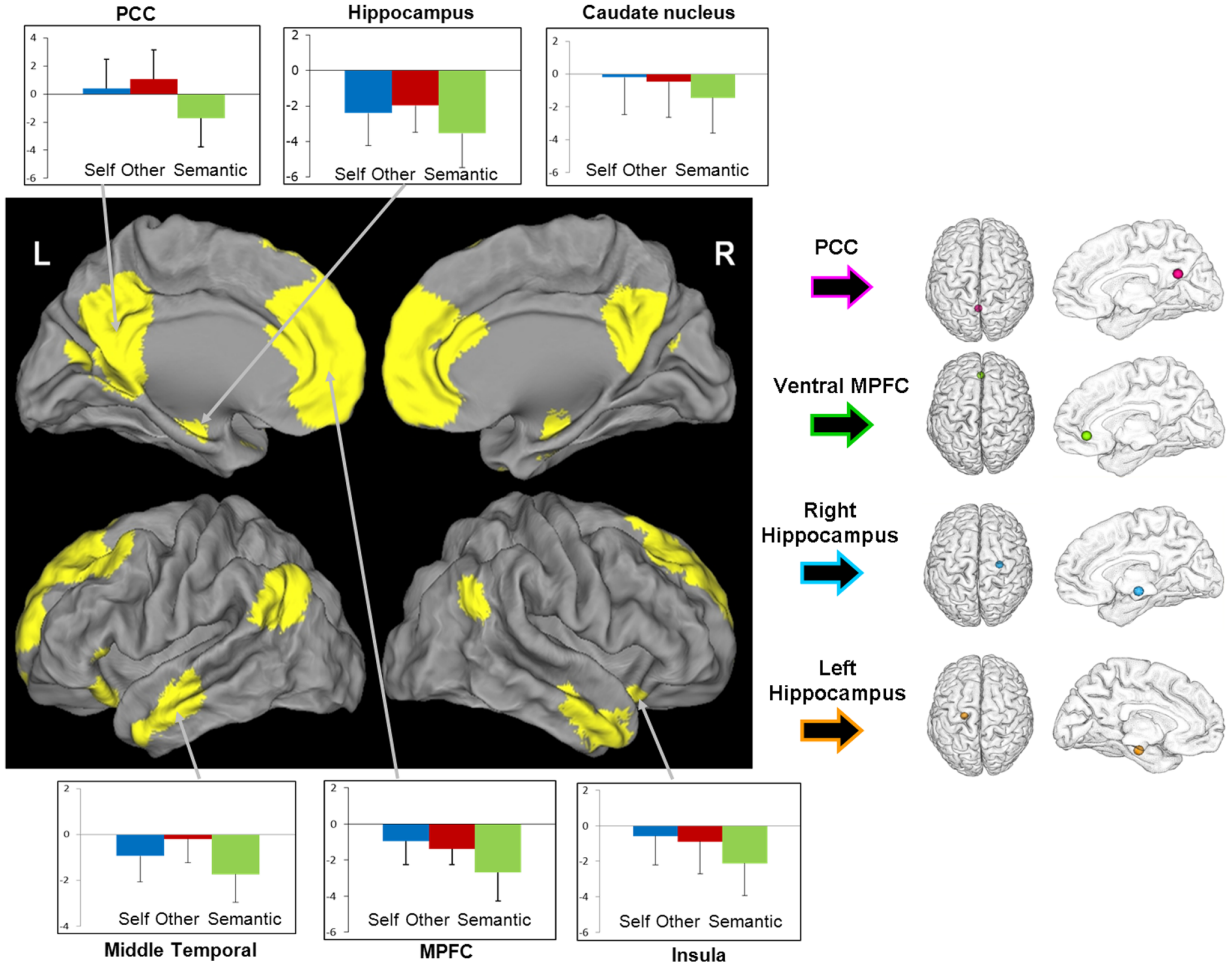
Brain activity changes associated with SRP during encoding (left panel). Results are displayed at p<0.005 uncorrected and k>50 voxels. L = Left, R = Right. The plots represent the mean BOLD value in the self, other and semantic conditions in each cluster of interest. The peaks located in the PCC, ventral MPFC, and left and right hippocampus from this analysis were used to create seeds for functional coupling analyses (right panel).

To further characterize the neural substrates of SRE, we performed similar analyses, this time assessing changes in brain functional coupling rather than changes in brain activity, using psychophysiological interaction (PPI) analyses. PPI analyses allow us to identify changes in functional coupling between seed regions and the rest of the brain that are driven by a self-reference psychological context [42], [43]. In the present study, the seed regions were chosen based on the results of the previous analyses as well as from *a priori* hypotheses based on existing literature. We thus selected regions that were highlighted in the previous analysis of SRP-related activity and that were of particular interest given our hypotheses (see above in the introduction), i.e. the PCC, the ventral MPFC and the hippocampus. These three regions were obtained in the analysis performed to highlight the brain activity associated with SRP during encoding so the coordinates of the center of the seeds used for the PPI analyses were obtained from these results ([−2 −58 22] for the PCC, [0 38 −6] for the ventral MPFC, [−22 −18 −22] for the left hippocampus and [28 −16 −12] for the right hippocampus; see Figure 3).

**Figure 4:**
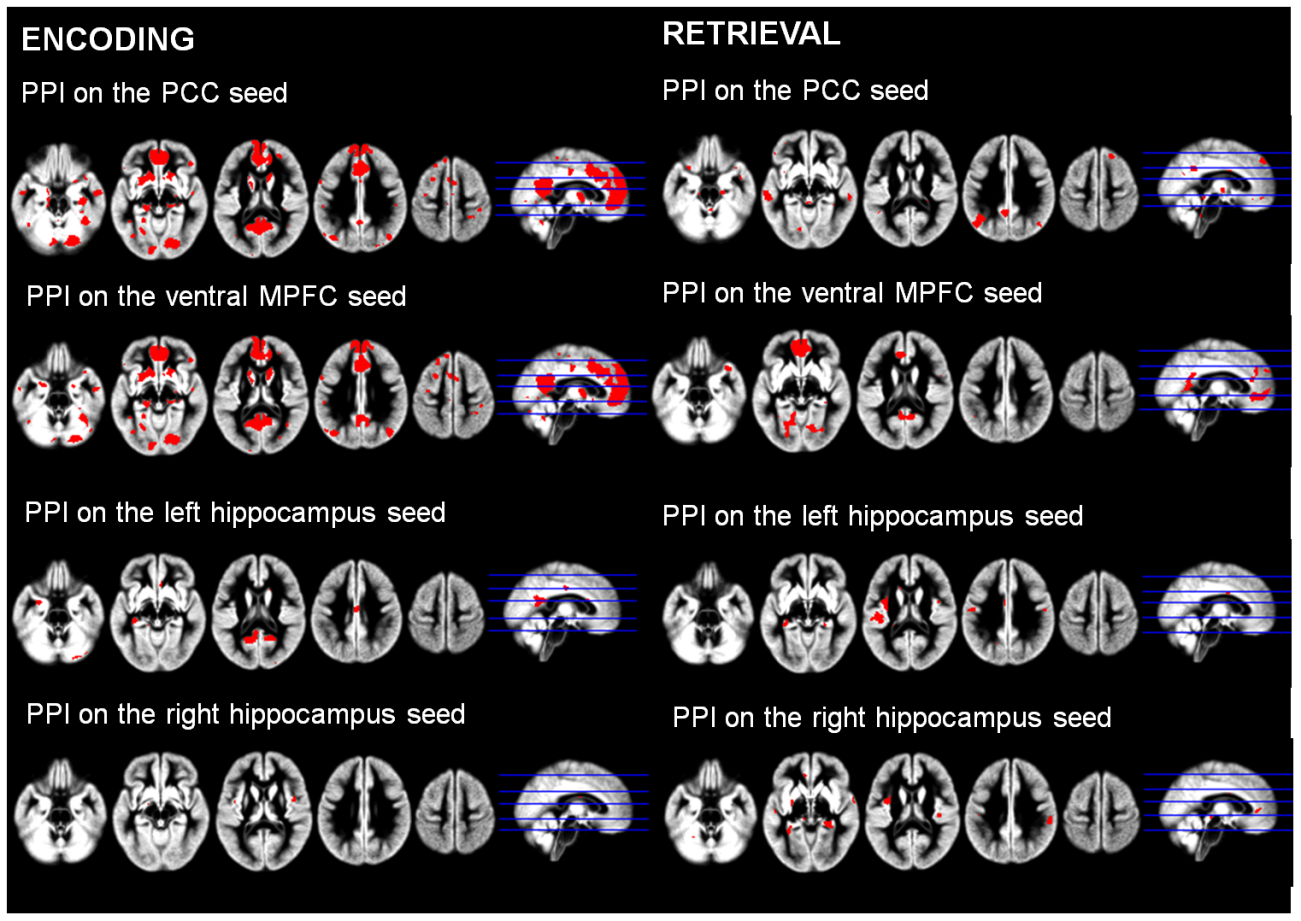
Brain functional coupling changes during successful encoding (left) and successful retrieval (right) of self-referential information. Brain functional coupling changes associated with the PCC (first line), ventral MPFC (second line), left (third line) and right (fourth line) hippocampus are displayed at p<0.05 uncorrected to be used as masks for analyses of SRE.

For each subject and for each seed, the neuronal activity for the contrast ‘Self minus Semantic’ of successfully remembered items was extracted for both encoding and recognition sessions from 6 spheres (of 6 mm radius) centered on the coordinates detailed above. Then, a linear model was built for each subject using three regressors. One regressor represented the successful retrieval modulated by the self-reference (Self Remembered) or semantic (Semantic Remembered) condition. The second regressor corresponded to the individual mean neuronal activity in each seed. The third (psychophysiological) regressor represented the interaction of interest between the first (psychological) and the second (physiological) regressors. The model also included movement parameters. Then, the same analysis and masking procedures as those described above to highlight brain activity associated with SRE were performed for each seed for both encoding and retrieval sessions. Thus, “group functional masks” corresponding to the 2^nd^ level one-sample analysis (thresholded at p uncorrected <0.05) on the individual images of the differences between ‘Self Remembered’ and ‘Self Forgotten’ items (obtained in 1^st^-level analyses) were computed for each of the four seeds and for both the encoding and the retrieval sessions (see Figure 4). Then, individual images of the differences between ‘Self Remembered’ and ‘Semantic Remembered’ items were computed in first level analyses, and entered in a second-level paired t-test analysis for each seed and both encoding and retrieval sessions, using the corresponding “group functional mask” as an inclusive mask.

Except for the “group functional masks” (see above), Results of all neuroimaging analyses were reported at p uncorrected <0.005 (and cluster extent k>50 voxels), with an indication on whether or not they survived at p (family wise error (FWE)) corrected <0.05 threshold.

## Results

### Behavioral results

Subjects correctly recognized 73.5% (±11.4%) of the adjectives seen during the self-reference condition and 62.1% (±13.4%) of those seen during the semantic processing control condition. Both retrieval percentages were significantly above random (i.e. 50%; one-sample t-test for Self retrieval: p = 10^−6^; one-sample t-test for Semantic retrieval: p = 3.10^−5^), and the paired t-test between the two conditions revealed a highly significant difference (self reference > semantic; p = 1.10^−6^).

### Neuroimaging results

#### Brain activity associated with Self-Reference Processing during encoding and retrieval

Encoding session: compared to the semantic processing, self-reference processing during encoding was associated with greater activation in various brain areas including the PCC, dorsal and ventral MPFC extending to the ACC, middle temporal cortex, insula, caudate nucleus and hippocampus, bilaterally (see Figure 3 and Table 1).

**Table 1:** Brain regions showing activity changes associated with SRP during encoding and retrieval.

Brain Region	k	t	MNI (peak)		
			x	y	z
**Self > Semantic during encoding**					
Posterior cingulate cortex (L)	3096	10*	−2	−58	22
Medial prefrontal cortex (L)	8387	9.62*	−8	62	16
Angular gyrus (L)	1085	7.42*	−48	−66	26
Middle temporal cortex (R)	889	6.50*	50	2	−30
Inferior frontal cortex (L)	496	6.19*	−30	24	−18
Caudate nucleus (L)	93	6.12*	−10	8	18
Middle temporal cortex (L)	828	6.02*	−60	−6	−16
Insula (R)	216	5.74	34	16	−16
Hippocampus (L)	184	5.68	−22	−18	−22
Hippocampus (R)	158	4.34	28	−16	−12
Angular gyrus (R)	479	4.47	56	−62	22
Inferior frontal cortex (L)	99	4.22	−46	22	6
Caudate nucleus (R)	52	4.07	10	8	18
Thalamus (L)	50	3.37	−2	−4	−4
**Self > Semantic during retrieval**					
Cerebellum (R)	114	3.57	34	−74	−20

Results are reported at p uncorrected <0.005 (and cluster extent k>50 voxels).

* FWE-corrected p value <0.05; k =  cluster size; t =  t-value; L = Left, R = Right.

Retrieval session: compared to the semantic processing, self-reference processing during retrieval was associated with greater activation in the cerebellum only (Table 1).

#### Brain activity associated with Self-Reference Effect during encoding and retrieval

**Figure 5:**
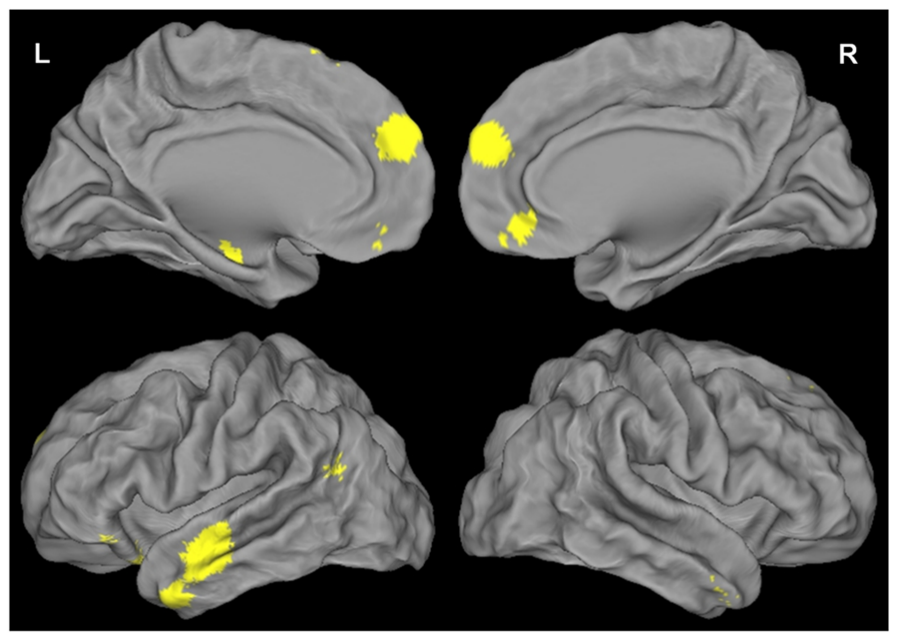
Brain activity changes associated with SRE during encoding. Results are displayed at p<0.005 uncorrected and k>50 voxels. L = Left, R = Right.

Encoding session: compared to the semantic condition, successful encoding of self-referential information was associated with greater activation in various brain areas including the dorsal and ventral MPFC extending to the ACC, left hippocampus, left insula and lateral temporal cortex bilaterally (see Figure 5 and Table 2).

**Table 2:** Brain regions showing activity changes associated with SRE during encoding and retrieval.

Brain Region	k	t	MNI (peak)		
			x	y	z
**Self Remembered > Semantic Remembered during encoding**					
Dorsal medial prefrontal cortex (R)	387	7.73*	4	56	20
Ventral medial prefrontal cortex (R)	90	7.58*	2	38	−6
Superior temporal gyrus (L)	147	6.46*	−50	4	−28
Middle temporal gyrus (L)	500	6.21*	−64	−16	−14
Superior temporal gyrus (R)	105	6.21*	52	10	−30
Superior frontal cortex (L)	87	5.59*	−6	18	66
Insula (L)	91	4.43*	−30	20	−16
Middle temporal gyrus (L)	90	4.89*	−40	−60	22
Superior frontal cortex (R)	50	4.69*	16	36	56
Hippocampus (L)	107	4.11	−26	−16	−26
Inferior frontal cortex (L)	92	4.10	−44	24	−12
**Self Remembered > Semantic Remembered during retrieval**					
No significant voxel					

Results are reported at p uncorrected <0.005 (and cluster extent k>50 voxels).

* FWE-corrected p value <0.05; k =  cluster size; t  = t-value; L =  Left, R =  Right.

Retrieval session: compared to the semantic condition, successful retrieval of self-referential information was not associated with increased activity in any brain area (Table 2).

#### Brain functional coupling associated with Self-Reference Effect during encoding and retrieval

**Figure 6:**
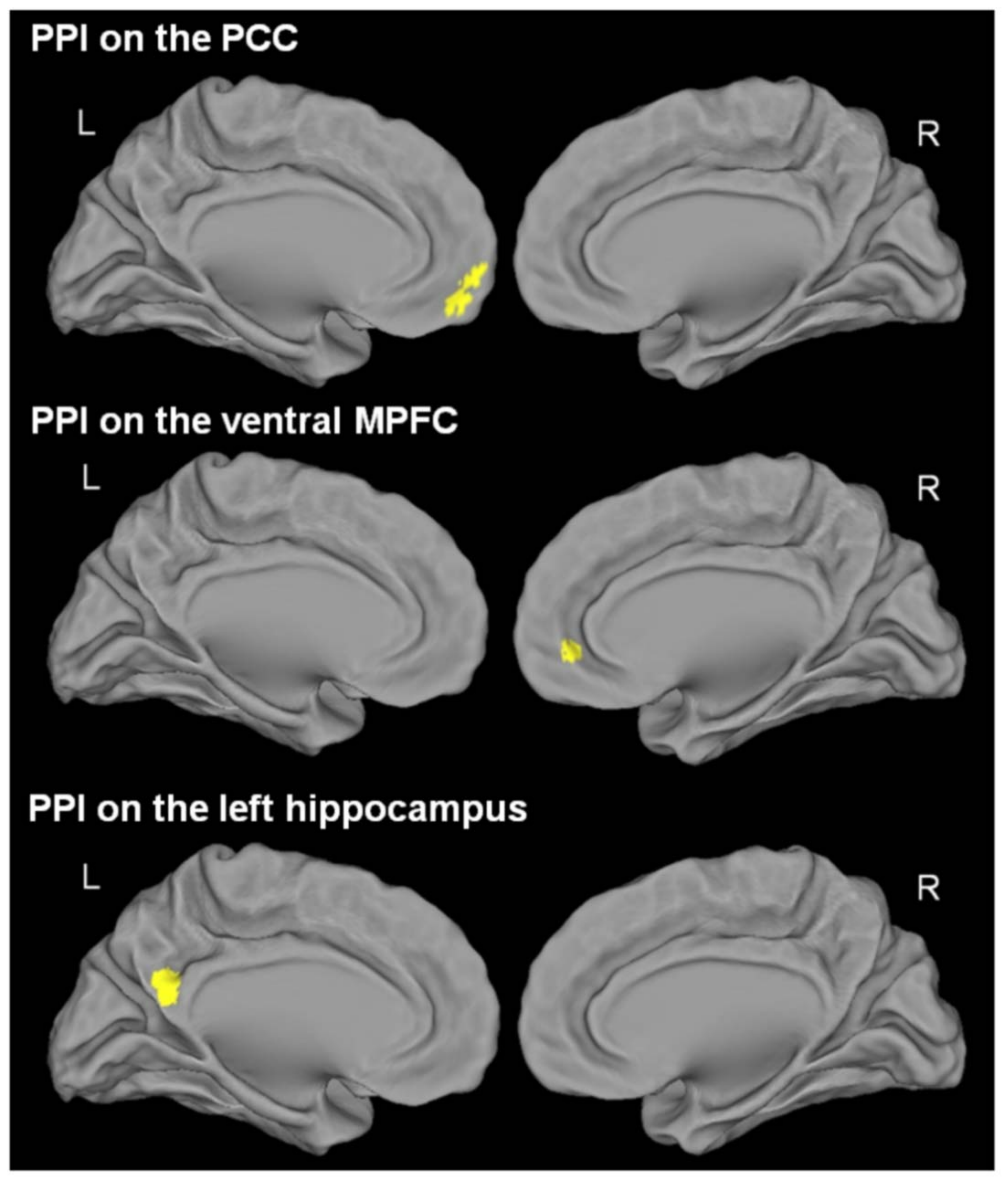
Brain functional coupling changes associated with SRE during encoding. The figure displays the regions showing greater functional coupling with the PCC (top), the ventral MPFC (middle), and the left hippocampus (bottom) during successful encoding of self-referential information compared to the corresponding semantic condition. The results are displayed at p<0.005 uncorrected and k>50 voxels. L = Left, R = Right.

Encoding session: compared to the semantic condition, successful encoding of self-referential information was associated with greater functional coupling between i) the PCC and the ventral MPFC, caudate nucleus and cerebellum; ii) the ventral MPFC and the ACC; iii) the left hippocampus and the PCC (see Figure 6 and Table 3). Note that the reciprocal connectivity increases (i.e. between the PCC seed and the left hippocampus, and between the ventral MPFC seed and the PCC) were recovered using a more permissive statistical threshold (p<0.05).

**Table 3:** Brain regions showing functional coupling changes with the PCC, the ventral MPFC and the hippocampus during successful encoding of self-referential items compared to the corresponding semantic condition.

Brain Region	k	t	MNI (peak)		
			x	y	z
**Self Remembered > Semantic Remembered during encoding**					
*PPI results from PCC seed*					
Ventral medial prefrontal cortex (L)	128	4.28	−12	56	0
Cerebellum (R)	70	3.62	16	−84	−32
Caudate nucleus (R)	50	3.57	20	16	16
*PPI results from ventral MPFC seed*					
Anterior cingulate cortex (R)	55	4.47	10	44	2
*PPI results from left hippocampus seed*					
Posterior cingulate cortex (L)	78	3.56	−8	−58	22
*PPI results from left hippocampus seed*					
No significant voxel					
**Self Remembered > Semantic Remembered during retrieval**					
*PPI results from PCC, ventral MPFC, and hippocampus seeds*					
No significant voxel					

Results are reported at p uncorrected <0.005 (and cluster extent k>50 voxels). None of the regions survived the threshold of FWE-corrected p<0.05; k =  cluster size; t =  t-value; L =  Left, R =  Right.

Retrieval session: as for brain activity, none of the PPI analyses revealed brain areas with significantly greater functional coupling associated with successful retrieval of self-referential information compared to the semantic condition (Table 3).

## Discussion

Our study shows that personality trait adjectives processed with self-reference are better remembered than words processed semantically, consistent with previous findings [15]–[18]. It also reveals that memory enhancement related to self-referential processing, called SRE, is associated with brain changes during the encoding phase only, including greater activity in the ventral MPFC and hippocampus, and greater functional coupling between these brain regions and the PCC.

### Encoding versus retrieval

The present study was designed to assess brain activity/connectivity changes underlying SRE (and SRP) during both encoding and retrieval so as to assess the relative contribution of both processes to SRE/SRP. Our findings showed that changes mainly occur during encoding. During retrieval, changes were restricted to the cerebellum for SRP, while there was no significant change for SRE. This suggests that SRE is not associated with the recruitment of additional brain regions/networks or increased connectivity between brain regions during retrieval. This is consistent with the findings of Sajonz et al. [44] who reported no significant SRE-related brain activity changes during retrieval, but in contrast with the involvement of the MPFC at retrieval reported in Benoit et al. [14]. However, the fact that item retrieval had to be associated with the context (condition) in which it was encoded (i.e. specifying, for each item to be recognized, whether it was previously seen in the self condition or not) may explain this difference as this may have promote the involvement of specific self-related processes during the retrieval of self-related information. Further studies are needed to better understand the potential implication of the MPFC in SRE during retrieval but our findings do suggest that SRE (as SRP) is associated with brain changes predominantly during encoding.

### Brain changes associated with SRP during encoding

In our study, SRP was associated with increased brain activity, compared to semantic processing, in a large network including the MPFC, PCC, lateral temporal cortex, hippocampus as well as insula and caudate nucleus. These findings are consistent with previous studies using the same experimental conditions (Self and Semantic judgments) [5]–[9]. Cortical midline structures, i.e. the MPFC and PCC, are considered as the basis for the “core self” as they are crucial to the processing of self-referential stimuli [45], [46]. More specifically, the MPFC (extending to the ACC) is known to be involved in evaluation (i.e. judgment of self-referential stimuli), representation (i.e. labeling of stimuli as self-referential), and monitoring of self-referential stimuli, while the PCC is thought to mediate the integration of self-relevant mental simulations with specific past experiences [35]–[39], [47], [48]. Moreover, the MPFC is considered as a key-component of the Self-Memory System (Conway, 2009) as, by contrast to other components involved in specific conditions only, the MPFC is activated in any condition related to the Self, i.e. from general self-knowledge to specific autobiographical memories. As for the hippocampus and lateral temporal cortex, both structures are also known to play a role in autobiographical memory retrieval, and more specifically in the episodic versus semantic components of autobiographical memory, respectively [49]. Also, together with the insula, both the MPFC and the PCC have been shown to be involved in the emotional judgment of self-referential words [6], [9], [50], [51]. Finally, it is interesting to note the similarity between the SRP encoding network and the Default-Mode Network (DMN) [52]–[55] which is thought to be involved in inner experience, introspection, self-related thoughts and autobiographical memory [52], [56]–[59].

### Brain changes associated with SRE during encoding

The main objective of the present study was to identify the neural basis of SRE, and we found changes, compared to a semantic processing condition, during the encoding session only. With regard to brain activity, increases were found in the MPFC, especially the ventral MPFC extending to the anterior cingulate cortex, hippocampus, insula and lateral temporal regions. The recruitment of the ventral MPFC during encoding of self-relevant information is consistent with previous studies [20], [21], [23], [60], [61] and further highlights the central role of this structure not only in episodic memory (i.e., learning of new information) and self-related processing, but also in the interaction between the processes that lead to SRE. Our study showed the recruitment of additional brain regions, i.e. the anterior cingulate cortex and the hippocampus, in line with previous reports [22], [23], [61], as well as the insula and lateral temporal cortex.

As mentioned above, the hippocampus and lateral temporal cortex are known to be involved in the retrieval of autobiographical memories, with hippocampal activity reflecting the retrieval of episodic details (sensory, perceptual, temporal) while the lateral temporal cortex is more specifically activated during semantic autobiographical memory tasks (general semantic knowledge retrieval) [62]–[66]. This overlap between the regions involved in autobiographical memory and those underlying SRE supports the hypothesis that SRE is at least partly subtended by the reactivation of self-related memories promoting the encoding of new information in episodic memory [4]. In other terms, the recollection of past personal events during the self judgment may promote the successful retrieval of self-related information.

The anterior cingulate cortex and the insula were found in both SRP and SRE during encoding in the present study. The anterior cingulate cortex is known to have a role in the monitoring of self-referential stimuli [45], while the insula is known to be involved in self-recognition [67]. The both structures have been shown to be involved in emotional processes [6], [50]. The recruitment of these regions may thus reflect the recollection of personal emotional events during SRP that would promote the successful retrieval of self-related information.

Over and above changes in terms of brain activity, we tested whether SRE was also underpinned by changes in terms of functional coupling during encoding or retrieval, and the present study is the first one to address this question. Again, our results revealed that brain functional coupling changes associated with the successful retrieval of self-related items were found during encoding, and not during retrieval. Interestingly, these changes mainly involved the connectivity between cortical midline structures, known to play a central role in self-related processing [45], and the hippocampus known as the main substrate of episodic memory on the other hand [12]. Our study thus suggests that, over and above increased activity, SRE is subserved by increased connectivity within and between the core self and episodic memory networks. This increased connectivity may lead to a deeper encoding of self-related items, a more specific trace with involvement of personal information and personal life experience, optimizing their subsequent retrieval. While the PCC activity did not change in relation to SRE, this structure showed reinforced connectivity with both the hippocampus and the ventral MPFC. Known as one of the main hubs in both the episodic memory and the self networks and to connect cortical midline structures to the medial temporal lobe [52], [68]–[70], the PCC appears as the best candidate to promote the communication between these structures and thereby to facilitate the encoding of new self-related information in episodic memory through the reactivation of self-related memories.

### Limitations

First, the use of a semantic condition as the reference task is open to criticisms. While this condition has been used as the reference in several previous studies ([8], [9] for example), other works have used the “Other” condition instead ([6], [7] for example). As explained in more details in the Method section, we considered that the other condition was not an optimal control condition to assess self-related processes because it is thought to involve self-relevant processes [8], [20], [34], [35]. This view is supported by the similitude between both associated brain networks (see Figure S1). However, it is worth noting that the semantic condition is not an optimal reference condition neither as it differs from the condition of interest not only by the self-oriented nature of the task, but also by the level of episodicity which is known to be related to the deepness of the processing [71]–[73]. Thus, the possibility that the self-related brain activity evidenced here also reflects the deeper level of processing associated with the episodic nature of the task cannot be excluded.

Second, the present study did not specifically assess the effect of the valence on SRE. Instead, the valence was counterbalanced and controlled for in all analyses. Indeed, our objectives were to assess brain activity and connectivity associated with SRP and SRE during encoding and retrieval, and further considering the valence as an additional variable of interest would have complicated the message and limited the statistical power in reducing the number of items per condition. Future studies may help clarifying the effect of the valence using different experimental or analysis designs.

Another limitation was the lack of significant results in the retrieval condition. As often when reporting negative findings, it is not possible to ascertain that subtle effects would not have been detected using a more sensitive method. However, the results of the present study suggest that brain activity and connectivity differences related to SRE are more substantial during encoding than during retrieval.

## Conclusion and Perspectives

This study provides insights into the brain changes associated with SRE, showing that changes occur mainly during encoding, include both increased brain activity and increased brain functional coupling in key brain areas for episodic memory and self-related processes. These findings in healthy young adults support the idea that the recollection of personal (autobiographical) life events during self-reference judgment promotes the successful retrieval of self-related information. Perspectives for future studies would include the consideration of the nature of the judgment (i.e. positive versus negative, and self-related versus other-related), as well as the self-relevance of the items (i.e. whether or not the items have been quoted as self-relevant by the subject) when assessing the brain substrates of SRE.

## Supporting Information

Figure S1 **Brain activity changes associated with SRP (red) and ORP (blue) during encoding.** The two pattern of activations greatly overlap (purple). Results are displayed at p<0.005 uncorrected and k>50 voxels.
(TIF)

Figure S2 **Global design of the statistical analyses and the corresponding masking procedure.** The procedure is illustrated for brain activity related to SRE during encoding, but the same was used for the corresponding analysis of the retrieval session and for the functional coupling analyses related to SRE during encoding and retrieval.
(TIF)
