# Supplementary figures and images for: Brain Activity and Functional Coupling Changes Associated with Self-Reference Effect during Both Encoding and Retrieval
Source: PLoS One. 2014 Mar 7;9(3):e90488. doi: 10.1371/journal.pone.0090488 (PMC3946483; doi:10.1371/journal.pone.0090488)

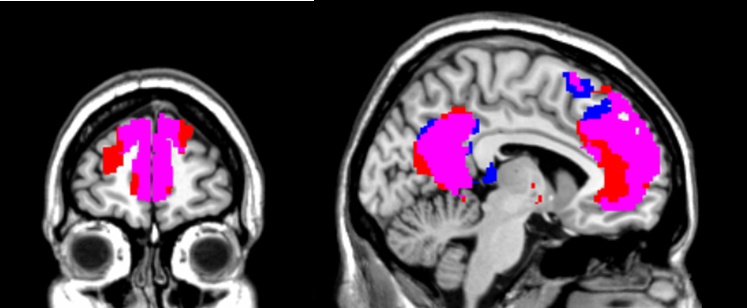

Supplement: Figure S1 — Brain activity changes associated with SRP (red) and ORP (blue) during encoding. The two pattern of activations greatly overlap (purple). Results are displayed at p<0.005 uncorrected and k>50 voxels. (TIF) [file pone.0090488.s001.tif]

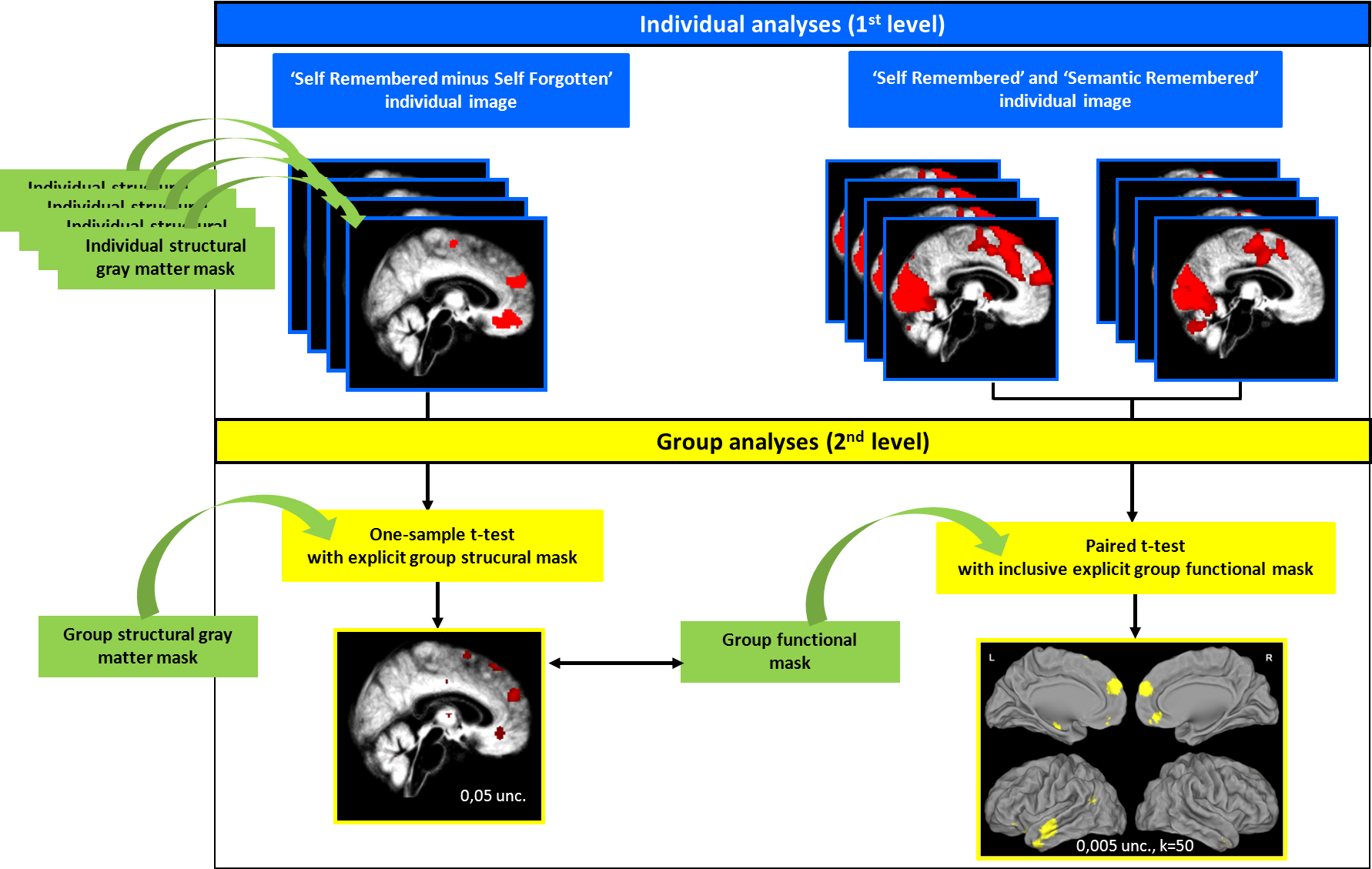

Supplement: Figure S2 — Global design of the statistical analyses and the corresponding masking procedure. The procedure is illustrated for brain activity related to SRE during encoding, but the same was used for the corresponding analysis of the retrieval session and for the functional coupling analyses related to SRE during encoding and retrieval. (TIF) [file pone.0090488.s002.tif]
